# Microglia: The Missing Link to Decipher and Therapeutically Control MS Progression?

**DOI:** 10.3390/ijms22073461

**Published:** 2021-03-27

**Authors:** Anastasia Geladaris, Darius Häusler, Martin S. Weber

**Affiliations:** 1 Department of Neuropathology, University Medical Center, 37075 Göttingen, Germany; anastasia.geladaris@med.uni-goettingen.de (A.G.); darius.haeusler@med.uni-goettingen.de (D.H.); 2 Department of Neurology, University Medical Center, 37075 Göttingen, Germany

**Keywords:** multiple sclerosis, disease progression, microglia, targets, therapy

## Abstract

Therapeutically controlling chronic progression in multiple sclerosis (MS) remains a major challenge. MS progression is defined as a steady loss of parenchymal and functional integrity of the central nervous system (CNS), occurring independent of relapses or focal, magnetic resonance imaging (MRI)-detectable inflammatory lesions. While it clinically surfaces in primary or secondary progressive MS, it is assumed to be an integral component of MS from the very beginning. The exact mechanisms causing progression are still unknown, although evolving evidence suggests that they may substantially differ from those driving relapse biology. To date, progression is assumed to be caused by an interplay of CNS-resident cells and CNS-trapped hematopoietic cells. On the CNS-resident cell side, microglia that are phenotypically and functionally related to cells of the monocyte/macrophage lineage may play a key role. Microglia function is highly transformable. Depending on their molecular signature, microglia can trigger neurotoxic pathways leading to neurodegeneration, or alternatively exert important roles in promoting neuroprotection, downregulation of inflammation, and stimulation of repair. Accordingly, to understand and to possibly alter the role of microglial activation during MS disease progression may provide a unique opportunity for the development of suitable, more effective therapeutics. This review focuses on the current understanding of the role of microglia during disease progression of MS and discusses possible targets for therapeutic intervention.

## 1. Introduction

Multiple sclerosis (MS) is the most common chronic inflammatory demyelinating disease of the central nervous system (CNS). There are four clinical MS subtypes defined: relapsing-remitting (RR), secondary-progressive (SP), primary-progressive (PP), and progressive-relapsing (PR) [1]. Relapses are associated with acute and/or ongoing focal inflammation, while progression is considered to reflect diffuse inflammation and neurodegenerative mechanisms. According to the new classification by Lublin et al., disease activity is defined by clinical relapses or the occurrence of gadolinium-enhancing lesions as well as new or unequivocally enlarging T2 lesions, while progression is defined as clinical deterioration in the absence of activity.

During progressive stages of MS, new lesions become less frequent, and progression is characterized by a steady increase in neurological disability, occurring independently of magnetic resonance imaging (MRI)-detectable focal inflammatory lesions [1].

For RRMS, huge progress has been made in the development of various disease-modifying therapies, which effectively reduce the number and the severity of new relapses as well as MRI activity [2]. However, most of the drugs are not designed and/or trailed to prevent disease progression. Until now, two drugs, ocrelizumab (Ocrevus) and siponimod (Mayzent), have shown therapeutic effects and have been approved for treatment of progressive MS forms. Although the approval was based on beneficial therapeutic results [3,4,5], only modest effects could be observed in the absence of Lublin-defined activity. One explanation might be that mechanisms that drive MS progression are distinct from the acute CNS infiltration of immune cells responsible for relapses in RRMS. While ocrelizumab as well as siponimod act mainly on this focal inflammatory component of the disease, which is not absent in PPMS or SPMS, their potential in limiting progression by itself remains insufficient. There are obvious differences between the relapsing and progressive stages of MS. Within the progressive forms, SPMS is distinguished from PPMS by its distinct disease course, which follows an initial course of RRMS. However, analyses of the pathology indicate that PPMS does not present different pathophysiological features from SPMS [6]. Importantly, several studies have shown that the early use of immunomodulatory drugs such as fingolimod, alemtuzumab, and natalizumab in RRMS patients have reduced the proportion of patients transitioning to SPMS, but had no direct influence on PPMS [7,8]. The conversion of RRMS to SPMS is thought to occur when the CNS exhausts its capacity to compensate for further axonal loss and recovery mechanisms are less effective [9]. Therefore, starting an early immunosuppression showed to be efficient in controlling disease activity and preventing irreversible damage. However, mechanism underlying progression are multifactorial and mainly characterized by neurodegeneration.

Therefore, therapeutic approaches that effectively target disease progression may have to focus on a different pathophysiological aspect of MS. Several mechanisms have been proposed to drive disease progression, including sustained compartmentalized inflammation behind a relatively closed blood–brain barrier (BBB) with continued involvement of hematopoietic cells and activation of CNS-resident cells such as microglia and astrocytes [10,11,12]. Microglia activation and microglia-driven neuroinflammation are considered as key events in the onset, progression, and resolution of MS. In the last years, the understanding of microglia function has grown and has generated major implications for therapeutic modulation of MS.

In this review, we summarize the current understanding of the mechanisms during disease progression with a particular focus on the role of microglia. Throughout the manuscript, we highlight promising treatment strategies by discussing possible therapeutic targets for halting or slowing MS progression.

## 2. Mechanism of Disease Progression in MS

The current knowledge regarding the mechanisms leading to disease progression includes chronic demyelination, gliosis, axonal loss, and an unbalance between damage and repair. As already mentioned, one major mechanism may be the interaction of CNS-established and CNS-resident cells. A number of observations make a contribution of T cells and B cells plausible. T cells were found in the cortical plaques of MS patients associated with disease progression, meningeal inflammation, and neurodegeneration [13]. Many of these T cell infiltrates are composed of CD8+ T cells with a phenotype of tissue resident memory cells, which show focally restricted activation [14]. One interaction partner of these CD8+ T cells was found to be mononuclear phagocytes composed of macrophages, microglia, and monocytes [15]. Furthermore, the presence of lymphoid follicle-like structures, memory B cells, and plasma cells in lesions and cerebrospinal fluid (CSF) of MS patients indicates that B cells can mature and perpetuate a compartmentalized, humoral immune response [16]. Interestingly, active demyelination and neurodegeneration can occur at a greater distance from T and B cell infiltrates [17]. Neuropathological studies have shown the presence of demyelination and axonal damage in the cortical and deep gray matter of MS patients, which are associated with microglial activation while lymphocytes are located in the meninges [18]. Therefore, it is likely that the activation of microglia is driven by soluble factors produced by T cells and/or B cells. Along the same lines, activated microglia and astrocytes can contribute to the persistence of B cells within the CNS by the secretion of specific molecules, such as BAFF and interleukin (IL)-6, which are known to support B cell survival [19]. Furthermore, disease progression is related to reduced repair and impaired axonal regeneration, in part due to age and a lifelong oxidative stress environment. Oligodendrocytes have especially been shown to be sensitive to oxidative stress, leading to an exhaustion in myelination capacity [20]. One result of demyelination and oligodendrocyte loss may be the activation of microglia and their production of reactive oxygen species (ROS) and nitric oxide (NO), which in turn provide a toxic environment and lead to axonal damage [10,11]. Of note, activated microglia interact also with astrocytes and therefore may limit the return to a homeostatic milieu. These mechanisms might explain the failure of peripherally acting MS drugs in preventing progression of MS.

### 2.1. Microglia—Heterogeneity and Plasticity

Microglia are the resident immune cells of the CNS [21,22]. They develop from immature yolk sac progenitor cells during early embryogenesis and persist throughout life [23]. Several studies highlight the plasticity and heterogeneity of microglia, which can switch between different phenotypes after CNS injury, including trauma, ischemia, and infection, and can participate in the maintenance of CNS homeostasis. Depending on the inflammatory milieu in various disease stages, microglia can differentiate into diverse phenotypes by changing their morphology, gene expression, and function [24]. Moreover, depending on their molecular signature, microglia can either trigger neurotoxic pathways leading to progressive neurodegeneration or exert important roles in promoting neuroprotection, downregulation of inflammation, and stimulation of repair [24]. The diverse microglia phenotypes were characterized by the presence of particular cell surface molecules and the expression of specific cytokines as well as chemokines, and have been classified into M1, M2a, M2b, or M2c subsets [25]. The classically activated M1 microglia phenotype is cytotoxic and exhibits pro-inflammatory markers, whereas the alternative M2 phenotype is divided into three different subtypes: M2a and M2b/c. M2a microglia are involved in repair and regeneration, M2b is associated with an immunoregulatory phenotype, and M2c with an acquired deactivating phenotype with repair and wound healing functions [26]. The homeostatic phenotype of microglia expresses several immune receptors, such as TREM2, SIRP1A, CXC3CR1, CSF-1R, and CD200R [23,27]. Furthermore, microglia express receptors that recognize damage-associated molecular pattern (DAMP) or neurodegeneration-associated molecular pattern (NAMP) molecules, which are released by injured and dying cells and can cause specific inflammatory responses. Upon stimulation, microglia downregulate some of these homeostatic genes while genes linked to phagocytosis, antigen presentation, and oxidative injury are upregulated [27]. In detail, potentially neurotoxic microglia, which promote inflammation and oligodendrocyte damage, present cell surface-expressed molecules such as MHCII and CD86, which allow T cells to recognize and bind small fragments of pathogens [28]. Furthermore, they produce pro-inflammatory mediators, such as NO, ROS, IL-1β, and tumor necrosis factor (TNF)-α [29,30]. In contrast, the neuroprotective M2 phenotype, which regulates immune functions and promotes repair, is characterized by increased phagocytosis and the production of diverse factors including arginase 1 (Arg1), CD206, insulin-like growth factor (IGF-2), and anti-inflammatory cytokines such as IL-10 [25]. However, the classification of these phenotypes likely to be over-simplified but at the same time a useful tool to study and understand the role of microglia in health and disease.

### 2.2. The Role of Microglia in MS Progression

Neuropathological studies from MS patients revealed an important role of chronically activated microglia during disease progression. Patients with a progressive disease course showed either chronic active (smoldering or expanding) lesions with microglial activation at the edge of a burned out plaque or inactive lesions with no microglial activity [31]. On the basis of the criteria first described by Luccinetti et al., one can subdivide active demyelination lesions into four different patterns (patterns I, II, III, and IV) [32]. Pattern I lesions show demyelination associated with activated macrophages/microglia, while in pattern II lesions complement activation is prominent, suggesting the involvement of antibodies. Pattern III lesions are characterized by the presence of oligodendrocytes with nuclear condensation and fragmentation, resembling apoptotic cell death. This is associated with a selective loss of myelin-associated glycoprotein, a myelin antigen located in the most distal (periaxonal) oligodendrocyte processes. Pattern IV lesions are exceptionally rare and show extensive non-apoptotic oligodendrocyte degeneration in the periplaque white matter adjacent to the active lesion, with limited repair and no evidence for either complement deposition or myelin-associated glycoprotein (MAG) loss [32,33]. Additional studies revealed increased numbers of microglia in active demyelinating lesions, which express markers associated with phagocytosis, oxidative injury, and antigen presentation or T cell co-stimulation, whereas no difference was found in microglia density between disease patterns [34]. In chronic demyelinating lesions, microglia change to a phenotype with both pro- and anti-inflammatory properties [34]. Furthermore, microglial activation was observed in areas surrounding the focal lesion, the so-called normal-appearing white matter (NAWM) [35,36]. 

Using positron emission tomography (PET) microglial activation was assessed in relapsing and progressive MS patients by measuring the mitochondrial translocator protein TSPO, which is upregulated in activated microglia [37]. It was observed that microglial activity correlates with disease disability and prognosis in progressive, but not with disability in relapsing MS patients, which could be associated with compartmentalized inflammation and neurodegeneration.

Using single-cell mass cytometry analysis, the phenotype of activated microglia in patients with progressive MS was determined. The study revealed that highly phagocytic and activated microglia downregulated the expression of homeostatic markers such as P2Y12 and GPR56 and upregulated the expression of proteins involved in phagocytic activity and microglial activation including CD68, CCR2, CD64, CD32, CD95, and CCL4 [38]. Besides the physical presence of microglia at sites of demyelination and an upregulation of various markers, the pathophysiological function of microglia in progression is largely unclear. During CNS inflammation, there is a marked increase in the expression of various pro-inflammatory cytokines (IL-6, IL-1β, C1q, and TNF-α) and chemokines (CCL2, CCL3, CCL4, and CCL5), both in MS and its animal model the experimental autoimmune encephalomyelitis (EAE) [39]. Microglia can produce numerous pro-inflammatory molecules, which may induce bystander effects to neighboring glial cells and neurons. For example, microglial TNF-α and C1q are involved in the induction of a neurotoxic A1 astrocyte phenotype, which can cause the rapid killing of both oligodendrocytes and neurons [40,41]. Due to their high metabolic activity, oligodendrocytes are especially susceptible to microglia-derived factors.

Furthermore, axonal damage is associated with mitochondrial injury, both in glial cells and neurons [11]. Mitochondrial injury in MS lesions can be enhanced by microglia and macrophages upon production of ROS and reactive nitrogen species (RNS) [42]. Notably, in patients with progressive MS, an increased number of neurons in the cerebral cortex are present with respiratory deficits [43]. NO can directly inhibit the mitochondrial respiratory chain complex IV as well as the cytochrome c oxidase (COX) and can thereby lead to axonal injury [44]. In addition, iron release from damaged oligodendrocytes can also contribute to oxidative stress. Although damaged oligodendrocytes are the major source of iron, it is assumed that microglia and macrophages take up this iron and undergo fragmentation and degeneration, thereby leading to a second release of iron, which induces axonal and neuronal destructions [45].

Microglia can also have neuroprotective function. Key functions, how microglia contribute to neuronal repair and therefore induce remyelination, are myelin debris clearance by phagocytosis and the production of anti-inflammatory cytokines such as IL-4, IL-10, and IL-13 [46,47]. Microglia have been shown to promote repair processes of damaged axons by phagocytosing of myelin debris in white matter (WM) lesions involving several receptors such as toll-like receptor (TLR), TREM2, CRs, FC, and PSR. These receptors are already known to be important in microglia phagocytosis [48]. For example, TREM2-deficient microglia fail to upregulate genes associated with phagocytosis and lipid metabolism [49]. Moreover, CX3CR1 knockout mice show reduced myelin debris clearance and impaired remyelination due to the ineffective function of microglia [47]. However, slowly expanding demyelination and the failure of remyelination may be one main mechanism of disease progression. Although the cause remains largely unknown, the lack of neuroprotective M2 microglia may be one explanation. Indeed, histopathological studies revealed a decreased number of oligodendrocyte precursor cells (OPCs) in lesions from patients with a progressive disease course, probably due to failure of OPC recruitment to the lesion site [50,51]. The recruitment of OPCs to the lesion site is benefitted by neuroprotective microglia as they clear debris and produce cytokines. Besides the induction of OPC migration, microglia polarization has been shown to play a role in oligodendroglial proliferation and differentiation. For example, a switch from a M1- to a M2-polarized phenotype was observed in a lysophosphatidylcholine (LPC)-mediated demyelination model during OPC proliferation at the initiation of remyelination [52]. In vitro experiments showed the ability of M2-conditioned media to promote OPC differentiation, whereas the depletion of M2 cells in focal demyelinating lesions prevented OPC differentiation. Finally, the blockade of activin-A secreted by M2 cells fully prevented OPC differentiation in demyelinated cerebellar slice cultures [52]. Other factors derived by astrocytes such as galectin-1 showed an induction of the M2 phenotype [53]. In detail, galectin-1 binds to M1 microglia, thereby modulating M1 key features, such as CCL2 and iNOS expression [53]. In addition, it was shown that the transcription factor homeobox protein MSX3 is associated with a microglia phenotype, which favors remyelination [54]. Importantly, the exact mechanism underlying a transition from pro-inflammatory to pro-regenerative microglia remains unknown. However, one possible mechanism is microglia death. A recent study showed that necroptosis of microglia is important in the phenotype transition during remyelination. The authors suggest that microglia may not switch between phenotypes, but pro-inflammatory microglia are dying in order to be replaced by alternative phenotypes [55]. In line with these findings, another study demonstrated the activation of RIPK1, RIPK3, and MLKL molecules, which are characteristic for necroptosis in MS lesions, in the cuprizone-induced demyelination model and in EAE [56]. RIPK1 is more highly expressed in activated microglia and to a lower extent in oligodendrocytes, and its inhibition prevents oligodendrocyte death [56]. Collectively, these observations suggest that microglia cell death might be essential for regeneration.

**Figure 1:**
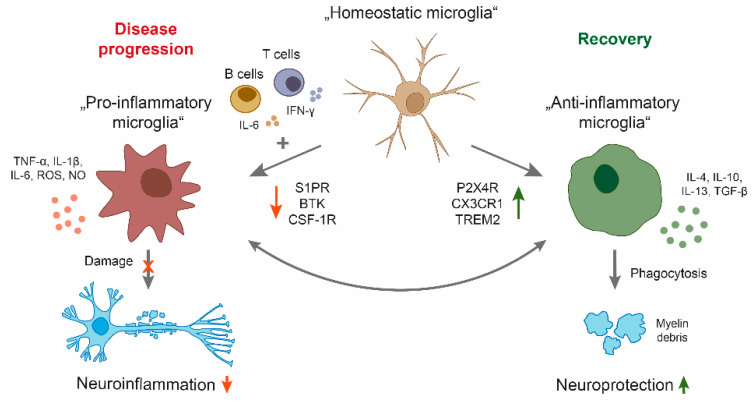
Schematic overview of microglia polarization in multiple sclerosis (MS). During disease progression, a central nervous system (CNS) compartmentalized milieu generated by pro-inflammatory B and T cells as well as CNS resident cells leads to a reactive phenotype of microglia with neuroinflammatory properties. By secreting cytokines and upregulation of particular cell surface molecules, the phenotype triggers oligodendrocyte damage, resulting in demyelination, axonal damage, and neuronal loss. On the other side, microglia have important physiological functions in maintaining tissue homeostasis, including clearance of debris, resulting in neuroprotection. Candidate drugs for treatment of MS progression may either inhibit pro-inflammatory (S1P, BTK, CSF-1R) or enhance anti-inflammatory properties of microglia (P2X4R, CX3CR1, TREM2). BTK: Bruton’s tyrosine kinase, CSF-1R: colony-stimulating factor 1 receptor, CX3CR1: CX3 chemokine receptor 1, IFNγ: Interferon-γ, IL: interleukin, NO: nitric oxide, P2X4R: purinergic receptor P2X4, ROS: reactive oxygen species, S1PR: sphingosine-1-phosphate receptor, TGF: transforming growth factor, TNF: tumor necrosis factor, TREM2: triggering receptor expressed on myeloid cells.

Given the different roles of microglia in regulating the pathology of MS, a balance between limiting demyelination and boosting remyelination may be a possible intervention as a long-term treatment strategy for patients with a progressive disease course (Figure 1). Another therapeutic approach could be the limitation of the neuroinflammatory properties of microglia. Indeed, microglia-based therapies have been mostly focused on the suppression of microglia-mediated inflammatory response and oxidative damage. Current research is focusing on anti-inflammatory molecules to reduce microglial activation in MS [57].

### 2.3. Biomarkers

To better understand the mechanisms underlying MS progression and to identify suitable therapies, researchers are investigating various biomarkers for the identification of MS progression. TSPO/PET is currently under investigation as a promising method to specifically use microglia activation as a biomarker for MS progression. Therefore, radioligands binding to the TSPO molecule are mostly used [58]. Studies of progressive MS have demonstrated an increase in TSPO uptake in the NAWM and NAGM, which appear to be related to disease severity and patient age [59]. In addition, PET imaging can also be used to differentiate between chronic active (smoldering) and chronic inactive lesions. In particular, the slowly expanding/smoldering lesions are thought to contribute to MS progression. Indeed, it was shown that in the brain of advanced SPMS patients, 57% of the plaques were of the chronic active type, with increased TSPO binding [60]. Moreover, an in vivo TSPO/PET study demonstrated that increased TSPO radioligand uptake in NAWM predicts later disability progression independent of relapse activity [61]. TSPO detects microglia activation but does not allow for the investigation of the different phenotypes. Therefore, other radioligands are under investigation binding, for example, the P2X7 purinergic receptor or iNOS, which are both associated with M1 microglia. However, due to the technical challenges and radiation exposure, TSPO/PET is not a widely used biomarker in clinical practice and there is a need for biomarkers that relate to MS progression that are more easily measurable. Another approach is measuring soluble molecules in the CSF and blood as promising potential biomarkers. CSF concentrations of several proteins have been suggested to reflect microglial activation in the brain, but most of these are also expressed by astrocytes, and therefore it has been difficult to distinguish microglia and astrocyte activation using biomarkers thus far. To our knowledge, one CSF protein has become established as a biomarker selective for microglial activation—the soluble form of the triggering receptor expressed on myeloid cells 2 (sTREM2). TREM2 is a cell surface receptor predominantly expressed on myeloid cells. It has been shown that the levels of sTREM2 are increased in patients with MS [62,63]. However, CSF levels of sTREM were found to be increased in all forms of MS and also in patients with other neurologic diseases, which suggests that sTREM2 is a general inflammatory marker rather than a disease-specific marker.

Extracellular vesicles (EVs) may be used as another source of potential biomarkers of MS disease stages and progression. EVs are lipid bilayer particles naturally released from cells, playing important roles in intracellular communications [64]. Compared to healthy controls, it has been shown that EVs are increased in the CSF of MS patients [65]. However, the amount of EVs was higher in active patients, suggesting that EVs display a marker for neuroinflammation rather than for disease progression. Overall inclusion of biomarkers associated with microglial activity and MS progression into the clinical diagnosis would enable a more individualized treatment possibility aiming to slow down MS progression.

## 3. Therapeutic Strategies to Stop MS Progression

Targeting microglial activity in MS progression by favoring the polarization of neuroprotective microglial phenotypes and simultaneously limiting neuroinflammation represents a promising therapeutic strategy (Figure 1). However, this remains challenging for multiple reasons. First, there is probably not one specific target that inhibits the pro-inflammatory microglial phenotype that at the same time induces a neuroprotective subset. Second, microglia cannot be easily accessed by therapeutics in the CNS. Although, in this review we mainly focus on microglia, the close interaction between microglia and other CNS resident cells such as astrocytes makes it difficult to specifically target microglia without inducing severe side effects by targeting surrounding cells.

### 3.1. Ocrelizumab—Targeting CNS-Established B Cells?

As mentioned above, one factor contributing to disease progression is the formation of B cell follicle-like structures in the meninges, which correlates with the development of cortical degeneration. The first approved drug to target PPMS is ocrelizumab. Ocrelizumab is an infusible humanized monoclonal antibody that selectively depletes CD20^+^ B cells. CD20 is expressed on B cells across different stages of maturation, ranging from pre-B cells in the bone marrow to short-lived plasmablasts, while long-lived antibody producing plasma cells completely downregulate CD20 expression. In a PPMS phase III study (ORATORIO), ocrelizumab significantly reduced the risk of disability progression and the rate of brain atrophy as compared to the placebo group. Furthermore, in a subgroup analysis, the effect of ocrelizumab was greater in younger patients but also in patients with increased disease activity, as identified by gadolinium-enhancing lesions [66]. Notably, the chimeric monoclonal antibody rituximab, which has a similar mechanism of action as ocrelizumab, did not reach the primary endpoint measure, the time to confirm disease progression in the OLYMPUS trial [67]. These differences probably result from trial design and patient population. However, a secondary analysis from the OLYMPUS trial showed that younger patients with evidence of focal inflammatory lesions have benefitted from the medication [67]. The results of both the ORATORIO and the OLYMPUS trials highlight the efficiency of B cell-depleting therapies in patients with an active disease course, suggesting that a peripheral B cell-targeting therapy is most efficient in disease stages characterized by acute inflammation.

### 3.2. The Sphingosine-1-Phosphate Receptor System

The modulation of sphingosine-1-phosphate receptor (S1PR) is an approved treatment for RRMS because of its anti-inflammatory effect of retaining lymphocytes in the lymph nodes, thus decreasing their entry into the CNS [68]. S1PRs belong to the G protein-coupled receptor family differentially expressed by various neuronal and peripheral cell populations, such as lymphocytes, dendritic cells, astrocytes, microglia, oligodendrocytes, and neurons. S1PRs are targeted by fingolimod and siponimod; however, only siponimod is approved for the treatment of SPMS, while fingolimod failed to reduce disability worsening in the INFORMS trial for PPMS [69,70]. Failure of fingolimod in the INFORMS trial may be linked to patient population and trial design. However, there are also several differences between fingolimod and siponimod. Fingolimod is a pro-drug that needs to be activated, whereas siponimod requires no activation. Furthermore, fingolimod binds to four of the five S1P receptors, while siponimod predominantly interferes with two receptor isoforms S1PR1 and S1PR5 [71].

The mechanism of action in the periphery of both drugs involves internalization of S1PRs in T cells, the subsequent attenuation of S1P-dependent transmigration of T cells out of lymph nodes, and a resulting reduction in aberrant autoimmune responses [68]. It was shown that both drugs can cross the BBB and, as already mentioned, S1PRs are also expressed by cells of the CNS [72]. It is therefore questionable as to whether the drug can have beneficial effects on MS progression by targeting CNS-resident cells directly or indirectly through the influence on peripheral T cells. In the EAE model, therapeutic siponimod treatment reduced EAE severity and diminished microglial MHC class II expression, but had no effect on the co-stimulatory molecule CD86 [73]. To investigate if the effect is mediated peripherally through T cells or centrally through CNS-resident cells, researchers administered siponimod directly into the brain using an intracerebroventricular infusion [74]. While this route of siponimod delivery also improved the severity of EAE, the number of peripheral CD3^+^ cells was not affected. In fact, microgliosis as well as astrogliosis were reduced after siponimod treatment. Still, it remains unclear as to whether the mode of action is mediated through CNS-established T cells or CNS-resident cells. A study focused on this issue using an organotypic slice culture model and found a reduced lysophosphatidylcholine (LPC)-mediated demyelination upon siponimod treatment [74]. Since peripheral immune cells are absent in the organotypic slice culture, this experimental setup has shown that siponimod can directly interact with CNS-resident cells. Moreover, a few in vitro studies investigated the direct interaction of siponimod with CNS-resident cells. For example, BV2 microglia showed a decreased release of IL-6 and CCL5 after siponimod treatment [75]. Furthermore, activated induced pluripotent astrocytes derived from stem cells, expressing S1PR, showed an ameliorated NFκB translocation, while at the same time Nrf2 nuclear translocation was enhanced after siponimod administration [76]. Astrocytic NFκB is primarily involved in pro-inflammatory reactions, scar formations, and neurodegeneration, while Nrf2 induces anti-oxidant, anti-inflammatory, and neuroprotective responses in vivo [77]. Whether there is a direct effect of siponimod on oligodendrocytes and neurons still remains unknown. However, a study using a *Xenopus* tadpole model showed an induced remyelination after siponimod treatment. The authors considered oligodendrocyte as the mediator because S1PR5 knockout in their model revealed no promotion of remyelination [78]. Importantly, S1PR5 is considered to be expressed only on oligodendrocytes within the brain.

Given the expression of various S1PRs on CNS-resident cells, targeting S1PR within the CNS by siponimod is an attractive approach to treat MS. However, the mechanism of action is probably an interaction of different effects on microglia, astrocytes, oligodendrocytes, and neurons. Moreover, it has to be kept in mind that siponimod especially attenuates the risk of disability progression in patients with ongoing inflammatory activity. Therefore, it remains unclear as to what extent progression itself is targeted in addition.

### 3.3. Modulating an Activating Enzyme: Bruton’s Tyrosine Kinase

Bruton’s tyrosine kinase (BTK), a member of the Tec family of kinases, is a cytoplasmic non-receptor tyrosine kinase expressed in cells of hematopoietic origin, including B cells, myeloid cells, and platelets, but not T or NK cells [79]. Besides its well-established mediation of BCR signaling, BTK is assumed to be involved in various signaling downstream to Fc, integrin, chemokine, and toll-like receptors [79,80]. Targeting B cells in MS is a well-approved treatment strategy; however, as already mentioned in Section 3.1, B cell-depleting antibodies show limitations of penetrating the BBB and modest results in slowing disease progression. To overcome these limitations, research has focused on BTK inhibition to target B cell activation. Moreover, due to the expression of BTK within the CNS, inhibition of BTK is a promising target strategy for the treatment of MS, including disease progression.

Evobrutinib, a selective BTK inhibitor, has already met its primary endpoint in the treatment of RRMS, defined as total number of T1 gadolinium-enhancing lesions in a phase II clinical trial. However, evobrutinib showed no effect on progression of disability [81]. Various other BTK inhibitors are being developed for the treatment of MS. The uniquely selective, noncovalent BTK inhibitor fenebrutinib is currently in a phase III trial in PPMS [82]. The ideal BTK inhibitor would be rapidly reversible, BBB-penetrant, and highly selective, and therefore could potentially reduce disease activity and slow disease progression. Notably, a reversible inhibitor, such as fenebrutinib, will probably need a relatively high CNS exposure to maintain therapeutic efficiency.

In general, BTK is activated by Lyn or Syk, leading to the activation of phospholipase Cγ (PLCγ) and to the promotion of Ca^2+^ influx [83]. Dysfunctional mutations of BTK cause the failure of B cell development, resulting in X-linked agammaglobulinemia in humans, a prototypic primary humoral immunodeficiency [84]. Moreover, deficiency in BTK or BTK inhibition alleviates Th17-cell-related inflammatory responses in various inflammatory mouse models. [85,86]. Within the CNS BTK is mainly expressed in microglia and to a lower extend in astrocytes [87]. The role of BTK in the CNS has been investigated in neuropathological studies, which showed an increased expression of BTK within lesions in progressive MS patients [88] and in demyelinating mouse models, independent of the adaptive immunity [86,88]. To reveal the direct mechanism of action, primary microglia were activated with complexed IgG, resulting in an induced BTK enzyme activity [88]. Moreover, inhibition of BTK with BTKi-1, a highly specific BTK inhibitor, has promoted remyelination in murine cerebellar slices ex vivo and in transgenic *Xenopus leavis* in vivo. The authors could not conclude whether the action of BTK inhibition on remyelination was mediated by microglia or astrocytes or both [86]. Studies using the less specific BTK inhibitor ibrutinib, which is approved for the treatment of certain cancers such as mantle cell or chronic lymphocytic leukemia, may help to understand the role of BTK in microglia and/or astrocytes [87,89]. Primary murine TLR4-induced microglia showed a reduction in TNF-α production after treatment with ibrutinib, whereas no effect was observed in IL-6 release [87]. In another study, inhibition of microglia or astrocytes with ibrutinib decreased the LPS-induced cytokine release of IL1-β, IL-6, and COX-2 in microglia but did not change the cytokine production in astrocytes [89]. Although the above-mentioned studies showed differences in their findings, which could result from the distinct cell culture model they used, they could highlight the potential of BTK inhibition in reducing the pro-inflammatory TLR-induced activation of microglia. One mode of action of BTK in the TLR signaling could be the activation of PLCy2 followed by calcium mobilization and activation of protein kinase C (PKC), NFκB, or NFAT [90]. A similar involvement of BTK is suggested in human microglia for the CCL5 signaling, in which BTK inhibition with the compound LFM-A13 resulted in a complete blockade of the CCL5-induced Ca^2+^ mobilization [91]. Thus far, it is not clearly understood as to which role BTK is playing in the various microglial signaling pathways. Moreover, it remains to be clarified whether BTK is mediating the neuro-inflammatory and/or neuro-protective properties.

### 3.4. Controlling Microglia Development and Maintenance: The CSF-1R System

The colony-stimulating factor 1 receptor (CSF-1R) is a cell surface receptor tyrosine kinase that binds to the ligands CSF-1 and IL-34 [92,93]. It is expressed on microglia, monocytes, and monocyte-derived cells [94]. The CSF-1R signaling directly controls microglial development as well as maintenance and autonomously regulates neuronal differentiation and survival [94]. In particular, CSF-1R signaling modulates proliferation, migration, differentiation, and survival of microglia and macrophages [95,96,97]. Loss-of function of CSF-1R causes hereditary diffuse leukoencephalopathy in humans [98]. Mice lacking CSF-1R exhibit reduced survival rates, and the number of microglia in *Csf1r*-null brains are reduced by more than 94% [99]. These results demonstrate the importance of CSF-1R in the development of microglia. Moreover, during inflammation the CSF-1R receptor is upregulated in several preclinical murine models of neuroinflammation and neurodegeneration as well as in the CNS tissue derived from progressive MS patients [100]. Treatment with a small-molecule ATP binding site inhibitor of the CSF-1R causes a rapid depletion of brain microglia in vivo, leading to a quick regeneration from brain-resident progenitor cells [95]. Additionally, a potent and selective CSF-1R inhibitor, which showed CNS penetrance, was able to inhibit CSF-1R dependent kinase phosphorylation, proliferation, and pro-inflammatory cytokine production in BV2 and primary murine microglia in vitro. Furthermore, therapeutic treatment with an inhibitor ameliorated the disease course of chronic-progressive, MOG peptide-induced, non-obese, diabetic (NOD) EAE [100]. Many other studies have shown the role of microglia in inflammation using a depleting dose of CSF-1R inhibitors [101,102,103,104]. For example, the inhibitor PLX5622 has shown an improvement in EAE severity by the depletion of microglia and macrophages [101]. These data suggest that inhibition of CSF-1R has an effect on the neuroinflammatory properties of microglia. Notably, these inhibitors also affect macrophages by reducing their number and by modulating their phenotype [102]. On the other hand, investigation of the involvement of microglia in de- and remyelination using the cuprizone model revealed that the blockade of CSF-1 by the inhibitors PLX3397 or BLZ945 resulted in decreased demyelination [103] and enhanced remyelination [104]. Importantly, it has been shown that under physiological conditions, a small number of neurons also express CSF-1R and excitotoxic injury results in increased CSF-1R expression [105]. However, whether neurons express CSF-1R in MS or EAE remains unknown. To our knowledge there are thus far no CSF-1R-modulating therapies in clinical trials for MS. Modeling CSF-1R may be beneficial in halting disease progression in MS by reducing inflammatory properties on microglia and by inducing neuroprotective effects. However, depletion of microglia using CSF-1R inhibition should be well timed and may only be efficient as a short-term treatment in combination with other medications.

### 3.5. TREM2: A Critical Modulator of Microglia Function

One critical modulator of microglial function is the triggering receptor expressed on myeloid cells 2 (TREM2), an innate immune receptor expressed on myeloid cells and exclusively on microglia in the brain. TREM2 associates with the adapter protein DNAX-activation protein 12 (DAP12) and DAP10, required for TREM2 surface expression and intracellular signaling [106]. Loss of function due to the mutation in TREM2 or DAP12 genes causes Nasu–Hakola disease, a rare genetic disorder, which is characterized by demyelination and microglial activation [107]. Other genetic variants in the receptor are associated with an increased risk for Alzheimer’s disease (AD), frontotemporal lobar degeneration (FTLD), or Parkinson’s disease (PD) [108,109]. The expression level of TREM2 varies depending on the CNS region, with a higher expression in the hippocampus, the spinal cord, and the white matter [110,111]. However, in vitro and in vivo data show contrary expression of TREM2 under inflammatory conditions. While anti-inflammatory molecules enhance TREM2 expression in vivo, pro-inflammatory molecules decrease TREM2 expression in vitro. Furthermore, it has been shown that its extracellular domain can be proteolyzed and is able to release the soluble form of TREM2 (sTREM2), which can function independently of TREM2 by regulating interactions between neurons and the surrounding microenvironment [112]. In the CSF of RRMS, SPMS, and PPMS patients, the level of sTREM2 was increased in comparison to healthy controls, and it is assumed that they indirectly reflect the expression or activity of the TREM2 receptor [63]. Various functions of TREM2 are known, e.g., primary murine microglia and macrophages deficient for TREM2 results in decreased phagocytosis of apoptotic neurons, cellular debris, and bacteria or bacteria products, while an increase in TREM2 expression enhances phagocytic activity [113]. Furthermore, knockout of TREM2 mice showed a defect in microglia myelin clearance in the cuprizone model [114]. In the same model, the treatment with a TREM2-agonistic antibody enhanced myelin debris clearance by microglia in vivo and by bone marrow-derived macrophages (BMDM) in vitro [115]. The enhanced phagocytosis recruited oligodendrocyte progenitor cells (OPC) and increased their differentiation into mature oligodendrocytes [115]. Therefore, targeting TREM2 in microglia could promote remyelination through the induction of microglial phagocytosis. In addition, TREM2 also modulates inflammatory signaling. TREM2 knockdown in microglia revealed an increased gene expression of TNF-α and NO synthase-2 transcription (NOS2), while overexpression of TREM2 decreased TNF-α, IL1-β, and NOS2 gene products [113]. However, some studies also have provided controversial results, e.g., TREM2 has been shown to be involved in promoting pro-inflammatory signaling both in mouse and human [116,117]. Both anti- and pro-inflammatory genes were found to be associated with TREM2 in the brain [111]. These findings highlight the complexity of TREM2 signaling in the brain. The challenge remains to find a compound that on one hand is able to increase microglial phagocytosis by targeting TREM2 and on the other hand may reduce inflammatory responses by sparing homeostatic or anti-inflammatory microglia.

### 3.6. CX3CR1 Expression on Microglia: A Switch towards an Inhibitory Phenotype?

CX3CR1 is a G-protein-coupled seven-transmembrane domain receptor in the CNS predominantly expressed on microglia [118]. It binds with high affinity to its ligand CX3CL1 (fractalkine), which is a chemokine existing in two forms. One form is membrane-bound and mainly expressed on neurons and endothelial cells, while the other form is a soluble form [119]. The functional outcome within the CNS of the CX3CR1/CX3CL1 axis seems to be restricted to microglia, where the signaling mediates a variety of microglial functions. Under homeostatic conditions, it is suggested that microglial activity is suppressed by CX3CR1/CX3CL1, as demonstrated in CX3CR1-deficient mice [120]. A higher level of microglial activity was accompanied by an increased neuronal death. Furthermore, it was shown that CX3CR1/CX3CL1 signaling participates in the control of production and release of several pro-inflammatory cytokines such as IL-1β, TNF-α, IL-6, and NO in LPS- and IFNγ-stimulated human and rodent microglia in vitro [121,122,123]. Other studies also showed the involvement of CX3CR1/CX3CL1 in the phagocytosis capacity of microglia [124,125].

In the EAE model, the expression of CX3CR1 was upregulated in lesions and sites of inflammation. The CX3CL1 ligand showed no changes in neurons but seemed to increase in astrocytes in the proximity of inflammation sites, which suggests that reactive or activated astrocytes may attract microglia to the sites of inflammation [126]. In fact, it has been demonstrated that CX3CR1/CX3CL1 also has an indirect effect on astrocytes by inducing the functional upregulation and increased expression of the excitatory amino acid transporter GLT-1 [127,128,129]. These data suggest a role for astrocytes in mediating neuroprotection induced by CX3CR1/CX3CL1. Moreover, mice lacking CX3CR1 showed a more severe EAE course and displayed overexpression of pro-inflammatory cytokines, i.e., TNF-α and higher levels of the anti-inflammatory cytokine IL-10 [130]. These results demonstrate the role of the CX3CR1/CX3CL1 axis in autoimmune regulation. As already described, microglia function has controversial roles—there is a large amount of evidence showing that microglia contribute to neuronal damage, but they also have important regenerative functions in MS. One hypothesis is that microglia themselves could upregulate CX3CL1-CX3CR1 expression and that this may be a mechanism by which microglia attempt to autoregulate their overactivation and return neighboring microglia to a quiescent state. This extent of autoregulation could push microglia toward neuronal destruction or to a more protective phenotype [131]. However, expression of CX3CR1 is not restricted to microglia, but it is also expressed in a subpopulation of monocytes, T cells, and NK cells [132]. It has been shown that CX3CR1 expression is upregulated in leukocytes of MS patients compared to healthy individuals [133]. A hallmark of RRMS is the infiltration of leukocytes into the CNS and CX3CR1, which has been shown to play a major role during this process. Therefore, functional inhibition of CX3CR1 is under investigation to prevent leukocyte infiltration into the brain [134]. Treating progression would require a different therapeutic strategy. A drug that favors expression of CX3CR1 in microglia may induce the anti-inflammatory microglial phenotype.

### 3.7. The Purinergic Receptor P2X4: Regulator towards Remyelination?

Cell damage causes high-grade ATP release. One receptor family that is rapidly activated by ATP is the P2X receptor family. In the CNS, the purinergic receptor P2X4 (P2X4R) is highly expressed in microglia and to a lower extent in neurons, oligodendrocytes, and astrocytes [135,136]. The receptor is associated with the homeostasis of major neurotransmission pathways. P2X4 knockout mice exhibit deficits in sensorimotor gating, social interactions, and ethanol drinking behavior [137,138].

The P2X4R plays a pivotal role on microglia chemotaxis and motility. For instance, it regulates the activation and migration of microglial cells at site of injury [135]. In MS patients and in EAE, P2X4R has been found to be upregulated [139]. A shift toward a P2X4R-expressing microglia phenotype is assumed to be regulated by the IRF8–IRF5 axis [140]. Previous studies have shown that the IRF8–IRF5 axis is involved in the polarization of pro-inflammatory microglia [141]. These results demonstrate that the expression of P2X4 in microglia may be associated with neuroinflammation.

In addition, P2X4 activation induces BDNF production and secretion, which modulates synaptic efficacy and accelerates OPC differentiation to mature oligodendrocytes and thus also favors remyelination [142,143]. These observations were confirmed by blocking P2X4R in microglia, which results in reduced oligodendrocyte differentiation and remyelination after exposure of a cerebellar slice culture to lysolecithin in vitro [144]. Due to the role of P2X4R in neuropathic pain after peripheral nerve injury, P2X4R blockers have already been proposed as potential therapeutic drugs for the treatment of neuropathic pain [145]. Conversely, an activation of P2X4R with the allosteric modulator ivermectin, which delays receptor deactivation, results in increased M2 microglia differentiation, phagocytosis of myelin debris, remyelination, and finally to an amelioration of EAE [144]. Although the main cell target for P2X4R modulators are microglia cells, expression in other CNS-resident cells, such as endothelial cells, Schwann cells, and in rare populations of neurons has been described [146]. Another study showed an increased activation and migration of CD4^+^ T cells after stimulation of P2X4R [147]. These facts have to be considered when testing P2X4R modulators for the treatment of MS. Nevertheless, modulation of the purinergic receptor is a potential candidate to promote the repair of myelin damage in MS.

## 4. Targeting Microglia Remains Challenging

Targeting microglia remains challenging for various reasons; evidence indicates that disease progression results from CNS-based inflammation behind an intact BBB. Therefore, drugs that target microglia have to first pass the BBB. The BBB is a complex barrier composed of a continuous layer of specialized endothelial cells linked together by tight junctions, surrounded by basal lamina, pericytes, and astrocytic endfeet [148]. This complex barrier regulates and limits the passage of molecules into and out of the CNS. During drug discovery, many tested compounds had failed due to lack of the ability to penetrate the BBB. Besides the size of the molecule, several strategies have been developed to overcome the BBB. For example, disruption of the barrier itself by osmotic or chemical agents. Another strategy is enhancing the transcellular transport of therapeutic agents. For this purpose, pro-drugs are tested, which only become pharmacologically activated either upon passing the BBB or upon microglia-specific interaction [149]. This may also limit the problem of off-target effects outside the CNS. In addition, the therapeutic agents would have to target microglia without inducing severe side effects by other CNS-resident cells. One promising option to deliver drugs specifically to microglia may be to pack drugs into nanoparticles, which are phagocytosed by microglia but not by any other cells in the CNS [150].

## 5. Conclusions

Microglia can have multiple roles in MS. In the context of progression, microglia are thought to be centrally involved in perpetuation of CNS-intrinsic inflammation. In our current understanding, microglia can produce pro-inflammatory cytokines and ROS, thereby causing axonal damage and neurodegeneration. The switch from a homeostatic- to a M1 phenotype is linked to changes in morphology and gene expression. On the basis of the molecular signature of microglia, researchers should develop therapeutics that can specifically and selectively suppress these neuroinflammatory properties while inducing neuroprotective functions. Here, we describe possible targets for such future interventions. We acknowledge that to date the generation of suitable drugs that can easily cross the BBB and furthermore accomplish this delicate task without inducing unwanted effects on other cells remains challenging. Nevertheless, in our mind, this general approach constitutes an extremely desirable goal to achieve, which in its accomplishment may initiate a new therapeutic era in MS, combining drugs halting progression with the established drugs preventing development of acute relapses.

## Data Availability

Not applicable to the study.

## References

[1] Lublin F.D., Reingold S.C., Cohen J.A., Cutter G.R., Sorensen P.S., Thompson A.J., Wolinsky J.S., Balcer L.J., Banwell B., Barkhof F. (2014). Defining the clinical course of multiple sclerosis: The 2013 revisions. Neurology.

[2] Costello F., Stuve O., Weber M.S., Zamvil S.S., Frohman E. (2007). Combination therapies for multiple sclerosis: Scientific rationale, clinical trials, and clinical practice. Curr. Opin. Neurol..

[3] U.S. Food and Drug Administration News Release (2018). FDA Approves New Drug to Treat Multiple Sclerosis. First Drug Approved for Primary Progressive MS. https://www.fda.gov/news-events/press-announcements/fda-approves-new-drug-treat-multiple-sclerosis.

[4] European Medicines Agency Press Release (2017). New Medicine for Multiple Sclerosis. https://www.ema.europa.eu/en/news/new-medicine-multiple-sclerosis.

[5] Rommer P.S., Milo R., Han M.H., Satyanarayan S., Sellner J., Hauer L., Illes Z., Warnke C., Laurent S., Weber M.S. (2019). Immunological Aspects of Approved MS Therapeutics. Front. Immunol..

[6] Correale J., Gaitan M.I., Ysrraelit M.C., Fiol M.P. (2017). Progressive multiple sclerosis: From pathogenic mechanisms to treatment. Brain.

[7] Brown J.W.L., Coles A., Horakova D., Havrdova E., Izquierdo G., Prat A., Girard M., Duquette P., Trojano M., Lugaresi A. (2019). Association of Initial Disease-Modifying Therapy With Later Conversion to Secondary Progressive Multiple Sclerosis. JAMA.

[8] Sucksdorff M., Tuisku J., Matilainen M., Vuorimaa A., Smith S., Keitila J., Rokka J., Parkkola R., Nylund M., Rinne J. (2019). Natalizumab treatment reduces microglial activation in the white matter of the MS brain. Neurol. Neuroimmunol. Neuroinflamm..

[9] Nave K.A., Trapp B.D. (2008). Axon-glial signaling and the glial support of axon function. Annu. Rev. Neurosci..

[10] Hochmeister S., Grundtner R., Bauer J., Engelhardt B., Lyck R., Gordon G., Korosec T., Kutzelnigg A., Berger J.J., Bradl M. (2006). Dysferlin is a new marker for leaky brain blood vessels in multiple sclerosis. J. Neuropathol. Exp. Neurol..

[11] Lisak R.P., Benjamins J.A., Bealmear B., Nedelkoska L., Studzinski D., Retland E., Yao B., Land S. (2009). Differential effects of Th1, monocyte/macrophage and Th2 cytokine mixtures on early gene expression for molecules associated with metabolism, signaling and regulation in central nervous system mixed glial cell cultures. J. Neuroinflamm..

[12] Abdelhak A., Weber M.S., Tumani H. (2017). Primary Progressive Multiple Sclerosis: Putting Together the Puzzle. Front. Neurol..

[13] Lucchinetti C.F., Popescu B.F., Bunyan R.F., Moll N.M., Roemer S.F., Lassmann H., Bruck W., Parisi J.E., Scheithauer B.W., Giannini C. (2011). Inflammatory cortical demyelination in early multiple sclerosis. N. Engl. J. Med..

[14] Smolders J., Heutinck K.M., Fransen N.L., Remmerswaal E.B.M., Hombrink P., Ten Berge I.J.M., van Lier R.A.W., Huitinga I., Hamann J. (2018). Tissue-resident memory T cells populate the human brain. Nat. Commun..

[15] Konjevic Sabolek M., Held K., Beltran E., Niedl A.G., Meinl E., Hohlfeld R., Lassmann H., Dornmair K. (2019). Communication of CD8(+) T cells with mononuclear phagocytes in multiple sclerosis. Ann. Clin. Transl. Neurol..

[16] Machado-Santos J., Saji E., Troscher A.R., Paunovic M., Liblau R., Gabriely G., Bien C.G., Bauer J., Lassmann H. (2018). The compartmentalized inflammatory response in the multiple sclerosis brain is composed of tissue-resident CD8+ T lymphocytes and B cells. Brain.

[17] Rommer P.S., Weber M.S., Illes Z., Zettl U.K. (2020). Editorial: Multiple Sclerosis—From Bench to Bedside: Currents Insights into Pathophysiological Concepts and Their Potential Impact on Patients. Front. Immunol..

[18] Magliozzi R., Howell O., Vora A., Serafini B., Nicholas R., Puopolo M., Reynolds R., Aloisi F. (2007). Meningeal B-cell follicles in secondary progressive multiple sclerosis associate with early onset of disease and severe cortical pathology. Brain.

[19] Krumbholz M., Theil D., Derfuss T., Rosenwald A., Schrader F., Monoranu C.M., Kalled S.L., Hess D.M., Serafini B., Aloisi F. (2005). BAFF is produced by astrocytes and up-regulated in multiple sclerosis lesions and primary central nervous system lymphoma. J. Exp. Med..

[20] Lassmann H., van Horssen J. (2016). Oxidative stress and its impact on neurons and glia in multiple sclerosis lesions. Biochim. Biophys. Acta.

[21] Perry V.H., Gordon S. (1988). Macrophages and microglia in the nervous system. Trends Neurosci..

[22] Kreutzberg G.W. (1996). Microglia: A sensor for pathological events in the CNS. Trends Neurosci..

[23] Ginhoux F., Lim S., Hoeffel G., Low D., Huber T. (2013). Origin and differentiation of microglia. Front. Cell Neurosci..

[24] Kettenmann H., Hanisch U.K., Noda M., Verkhratsky A. (2011). Physiology of microglia. Physiol. Rev..

[25] Martinez F.O., Sica A., Mantovani A., Locati M. (2008). Macrophage activation and polarization. Front. Biosci..

[26] Chhor V., Le Charpentier T., Lebon S., Ore M.V., Celador I.L., Josserand J., Degos V., Jacotot E., Hagberg H., Savman K. (2013). Characterization of phenotype markers and neuronotoxic potential of polarised primary microglia in vitro. Brain Behav. Immun..

[27] Aguzzi A., Barres B.A., Bennett M.L. (2013). Microglia: Scapegoat, saboteur, or something else?. Science.

[28] Schetters S.T.T., Gomez-Nicola D., Garcia-Vallejo J.J., Van Kooyk Y. (2017). Neuroinflammation: Microglia and T Cells Get Ready to Tango. Front. Immunol..

[29] Ding A.H., Nathan C.F., Stuehr D.J. (1988). Release of reactive nitrogen intermediates and reactive oxygen intermediates from mouse peritoneal macrophages. Comparison of activating cytokines and evidence for independent production. J. Immunol..

[30] Colton C.A., Gilbert D.L. (1987). Production of superoxide anions by a CNS macrophage, the microglia. FEBS Lett..

[31] Frischer J.M., Bramow S., Dal-Bianco A., Lucchinetti C.F., Rauschka H., Schmidbauer M., Laursen H., Sorensen P.S., Lassmann H. (2009). The relation between inflammation and neurodegeneration in multiple sclerosis brains. Brain.

[32] Lucchinetti C., Bruck W., Parisi J., Scheithauer B., Rodriguez M., Lassmann H. (2000). Heterogeneity of multiple sclerosis lesions: Implications for the pathogenesis of demyelination. Ann. Neurol..

[33] Kuhlmann T., Ludwin S., Prat A., Antel J., Bruck W., Lassmann H. (2017). An updated histological classification system for multiple sclerosis lesions. Acta Neuropathol..

[34] Zrzavy T., Hametner S., Wimmer I., Butovsky O., Weiner H.L., Lassmann H. (2017). Loss of ’homeostatic’ microglia and patterns of their activation in active multiple sclerosis. Brain.

[35] De Groot C.J., Bergers E., Kamphorst W., Ravid R., Polman C.H., Barkhof F., van der Valk P. (2001). Post-mortem MRI-guided sampling of multiple sclerosis brain lesions: Increased yield of active demyelinating and (p)reactive lesions. Brain.

[36] Van der Poel M., Ulas T., Mizee M.R., Hsiao C.C., Miedema S.S.M., Adelia, Schuurman, K (2019). G.; Helder, B.; Tas, S.W.; Schultze, J.L.; et al. Transcriptional profiling of human microglia reveals grey-white matter heterogeneity and multiple sclerosis-associated changes. Nat. Commun..

[37] Giannetti P., Politis M., Su P., Turkheimer F., Malik O., Keihaninejad S., Wu K., Reynolds R., Nicholas R., Piccini P. (2014). Microglia activation in multiple sclerosis black holes predicts outcome in progressive patients: An in vivo [(11)C](R)-PK11195-PET pilot study. Neurobiol. Dis..

[38] Bottcher C., van der Poel M., Fernandez-Zapata C., Schlickeiser S., Leman J.K.H., Hsiao C.C., Mizee M.R., Adelia, Vincenten, M (2020). C.J.; Kunkel, D.; et al. Single-cell mass cytometry reveals complex myeloid cell composition in active lesions of progressive multiple sclerosis. Acta Neuropathol. Commun..

[39] O’Loughlin E., Madore C., Lassmann H., Butovsky O. (2018). Microglial Phenotypes and Functions in Multiple Sclerosis. Cold Spring Harb. Perspect. Med..

[40] Liddelow S.A., Guttenplan K.A., Clarke L.E., Bennett F.C., Bohlen C.J., Schirmer L., Bennett M.L., Munch A.E., Chung W.S., Peterson T.C. (2017). Neurotoxic reactive astrocytes are induced by activated microglia. Nature.

[41] Rothhammer V., Borucki D.M., Tjon E.C., Takenaka M.C., Chao C.C., Ardura-Fabregat A., de Lima K.A., Gutierrez-Vazquez C., Hewson P., Staszewski O. (2018). Microglial control of astrocytes in response to microbial metabolites. Nature.

[42] Fischer M.T., Sharma R., Lim J.L., Haider L., Frischer J.M., Drexhage J., Mahad D., Bradl M., van Horssen J., Lassmann H. (2012). NADPH oxidase expression in active multiple sclerosis lesions in relation to oxidative tissue damage and mitochondrial injury. Brain.

[43] Campbell G.R., Ziabreva I., Reeve A.K., Krishnan K.J., Reynolds R., Howell O., Lassmann H., Turnbull D.M., Mahad D.J. (2011). Mitochondrial DNA deletions and neurodegeneration in multiple sclerosis. Ann. Neurol..

[44] Mahad D.J., Ziabreva I., Campbell G., Lax N., White K., Hanson P.S., Lassmann H., Turnbull D.M. (2009). Mitochondrial changes within axons in multiple sclerosis. Brain.

[45] Hametner S., Wimmer I., Haider L., Pfeifenbring S., Bruck W., Lassmann H. (2013). Iron and neurodegeneration in the multiple sclerosis brain. Ann. Neurol..

[46] Karamita M., Barnum C., Mobius W., Tansey M.G., Szymkowski D.E., Lassmann H., Probert L. (2017). Therapeutic inhibition of soluble brain TNF promotes remyelination by increasing myelin phagocytosis by microglia. JCI Insight.

[47] Lampron A., Larochelle A., Laflamme N., Prefontaine P., Plante M.M., Sanchez M.G., Yong V.W., Stys P.K., Tremblay M.E., Rivest S. (2015). Inefficient clearance of myelin debris by microglia impairs remyelinating processes. J. Exp. Med..

[48] Yamasaki R., Lu H., Butovsky O., Ohno N., Rietsch A.M., Cialic R., Wu P.M., Doykan C.E., Lin J., Cotleur A.C. (2014). Differential roles of microglia and monocytes in the inflamed central nervous system. J. Exp. Med..

[49] Poliani P.L., Wang Y., Fontana E., Robinette M.L., Yamanishi Y., Gilfillan S., Colonna M. (2015). TREM2 sustains microglial expansion during aging and response to demyelination. J. Clin. Investig..

[50] Boyd A., Zhang H., Williams A. (2013). Insufficient OPC migration into demyelinated lesions is a cause of poor remyelination in MS and mouse models. Acta Neuropathol..

[51] Franklin R.J., Goldman S.A. (2015). Glia Disease and Repair-Remyelination. Cold Spring Harb. Perspect. Biol..

[52] Miron V.E., Boyd A., Zhao J.W., Yuen T.J., Ruckh J.M., Shadrach J.L., van Wijngaarden P., Wagers A.J., Williams A., Franklin R.J.M. (2013). M2 microglia and macrophages drive oligodendrocyte differentiation during CNS remyelination. Nat. Neurosci..

[53] Starossom S.C., Mascanfroni I.D., Imitola J., Cao L., Raddassi K., Hernandez S.F., Bassil R., Croci D.O., Cerliani J.P., Delacour D. (2012). Galectin-1 deactivates classically activated microglia and protects from inflammation-induced neurodegeneration. Immunity.

[54] Yu Z., Sun D., Feng J., Tan W., Fang X., Zhao M., Zhao X., Pu Y., Huang A., Xiang Z. (2015). MSX3 Switches Microglia Polarization and Protects from Inflammation-Induced Demyelination. J. Neurosci..

[55] Lloyd A.F., Davies C.L., Holloway R.K., Labrak Y., Ireland G., Carradori D., Dillenburg A., Borger E., Soong D., Richardson J.C. (2019). Central nervous system regeneration is driven by microglia necroptosis and repopulation. Nat. Neurosci..

[56] Ofengeim D., Ito Y., Najafov A., Zhang Y., Shan B., DeWitt J.P., Ye J., Zhang X., Chang A., Vakifahmetoglu-Norberg H. (2015). Activation of necroptosis in multiple sclerosis. Cell Rep..

[57] Djedovic N., Stanisavljevic S., Jevtic B., Momcilovic M., Lavrnja I., Miljkovic D. (2017). Anti-encephalitogenic effects of ethyl pyruvate are reflected in the central nervous system and the gut. Biomed. Pharmacother..

[58] Airas L., Nylund M., Rissanen E. (2018). Evaluation of Microglial Activation in Multiple Sclerosis Patients Using Positron Emission Tomography. Front. Neurol..

[59] Herranz E., Gianni C., Louapre C., Treaba C.A., Govindarajan S.T., Ouellette R., Loggia M.L., Sloane J.A., Madigan N., Izquierdo-Garcia D. (2016). Neuroinflammatory component of gray matter pathology in multiple sclerosis. Ann. Neurol..

[60] Rissanen E., Tuisku J., Rokka J., Paavilainen T., Parkkola R., Rinne J.O., Airas L. (2014). In Vivo Detection of Diffuse Inflammation in Secondary Progressive Multiple Sclerosis Using PET Imaging and the Radioligand (1)(1)C-PK11195. J. Nucl. Med..

[61] Sucksdorff M., Matilainen M., Tuisku J., Polvinen E., Vuorimaa A., Rokka J., Nylund M., Rissanen E., Airas L. (2020). Brain TSPO-PET predicts later disease progression independent of relapses in multiple sclerosis. Brain.

[62] Piccio L., Buonsanti C., Cella M., Tassi I., Schmidt R.E., Fenoglio C., Rinker J., Naismith R.T., Panina-Bordignon P., Passini N. (2008). Identification of soluble TREM-2 in the cerebrospinal fluid and its association with multiple sclerosis and CNS inflammation. Brain.

[63] Ohrfelt A., Axelsson M., Malmestrom C., Novakova L., Heslegrave A., Blennow K., Lycke J., Zetterberg H. (2016). Soluble TREM-2 in cerebrospinal fluid from patients with multiple sclerosis treated with natalizumab or mitoxantrone. Mult. Scler..

[64] Budnik V., Ruiz-Canada C., Wendler F. (2016). Extracellular vesicles round off communication in the nervous system. Nat. Rev. Neurosci..

[65] Verderio C., Muzio L., Turola E., Bergami A., Novellino L., Ruffini F., Riganti L., Corradini I., Francolini M., Garzetti L. (2012). Myeloid microvesicles are a marker and therapeutic target for neuroinflammation. Ann. Neurol..

[66] Montalban X., Hauser S.L., Kappos L., Arnold D.L., Bar-Or A., Comi G., de Seze J., Giovannoni G., Hartung H.P., Hemmer B. (2017). Ocrelizumab versus Placebo in Primary Progressive Multiple Sclerosis. N. Engl. J. Med..

[67] Hawker K., O’Connor P., Freedman M.S., Calabresi P.A., Antel J., Simon J., Hauser S., Waubant E., Vollmer T., Panitch H. (2009). Rituximab in patients with primary progressive multiple sclerosis: Results of a randomized double-blind placebo-controlled multicenter trial. Ann. Neurol..

[68] Matloubian M., Lo C.G., Cinamon G., Lesneski M.J., Xu Y., Brinkmann V., Allende M.L., Proia R.L., Cyster J.G. (2004). Lymphocyte egress from thymus and peripheral lymphoid organs is dependent on S1P receptor 1. Nature.

[69] Kappos L., Bar-Or A., Cree B.A.C., Fox R.J., Giovannoni G., Gold R., Vermersch P., Arnold D.L., Arnould S., Scherz T. (2018). Siponimod versus placebo in secondary progressive multiple sclerosis (EXPAND): A double-blind, randomised, phase 3 study. Lancet.

[70] Lublin F., Miller D.H., Freedman M.S., Cree B.A.C., Wolinsky J.S., Weiner H., Lubetzki C., Hartung H.P., Montalban X., Uitdehaag B.M.J. (2016). Oral fingolimod in primary progressive multiple sclerosis (INFORMS): A phase 3, randomised, double-blind, placebo-controlled trial. Lancet.

[71] Kipp M. (2020). Does Siponimod Exert Direct Effects in the Central Nervous System?. Cells.

[72] Foster C.A., Howard L.M., Schweitzer A., Persohn E., Hiestand P.C., Balatoni B., Reuschel R., Beerli C., Schwartz M., Billich A. (2007). Brain penetration of the oral immunomodulatory drug FTY720 and its phosphorylation in the central nervous system during experimental autoimmune encephalomyelitis: Consequences for mode of action in multiple sclerosis. J. Pharmacol. Exp. Ther..

[73] Husseini L., Geladaris A., Steinleitner M., Grondey K., Koch J., Häusler D., Weber M. (2020). Siponimod treatment leads to a dose-dependent reduction of EAE severity associated with downregulation of microglial activity. MSVirtual.

[74] O’Sullivan C., Schubart A., Mir A.K., Dev K.K. (2016). The dual S1PR1/S1PR5 drug BAF312 (Siponimod) attenuates demyelination in organotypic slice cultures. J. Neuroinflamm..

[75] Gentile A., Musella A., Bullitta S., Fresegna D., De Vito F., Fantozzi R., Piras E., Gargano F., Borsellino G., Battistini L. (2016). Siponimod (BAF312) prevents synaptic neurodegeneration in experimental multiple sclerosis. J. Neuroinflamm..

[76] Colombo E., Bassani C., De Angelis A., Ruffini F., Ottoboni L., Comi G., Martino G., Farina C. (2020). Siponimod (BAF312) Activates Nrf2 While Hampering NFkappaB in Human Astrocytes, and Protects From Astrocyte-Induced Neurodegeneration. Front. Immunol..

[77] Draheim T., Liessem A., Scheld M., Wilms F., Weissflog M., Denecke B., Kensler T.W., Zendedel A., Beyer C., Kipp M. (2016). Activation of the astrocytic Nrf2/ARE system ameliorates the formation of demyelinating lesions in a multiple sclerosis animal model. Glia.

[78] Mannioui A., Vauzanges Q., Fini J.B., Henriet E., Sekizar S., Azoyan L., Thomas J.L., Pasquier D.D., Giovannangeli C., Demeneix B. (2018). The Xenopus tadpole: An in vivo model to screen drugs favoring remyelination. Mult. Scler..

[79] Hendriks R.W., Yuvaraj S., Kil L.P. (2014). Targeting Bruton’s tyrosine kinase in B cell malignancies. Nat. Rev. Cancer.

[80] Lopez-Herrera G., Vargas-Hernandez A., Gonzalez-Serrano M.E., Berron-Ruiz L., Rodriguez-Alba J.C., Espinosa-Rosales F., Santos-Argumedo L. (2014). Bruton’s tyrosine kinase—An integral protein of B cell development that also has an essential role in the innate immune system. J. Leukoc. Biol..

[81] Montalban X., Arnold D.L., Weber M.S., Staikov I., Piasecka-Stryczynska K., Willmer J., Martin E.C., Dangond F., Syed S., Wolinsky J.S. (2019). Placebo-Controlled Trial of an Oral BTK Inhibitor in Multiple Sclerosis. N. Engl. J. Med..

[82] Gheen M., Hauser S., Bar-Or A., Francis G., Giovannoni G., Kappos L., Nicholas J., Oh J., Sormani M.P., Stoll S. (2020). Examination of fenebrutinib, a highly selective BTKi, on disease progression of multiple sclerosis. MSVirtual.

[83] Humphries L.A., Dangelmaier C., Sommer K., Kipp K., Kato R.M., Griffith N., Bakman I., Turk C.W., Daniel J.L., Rawlings D.J. (2004). Tec kinases mediate sustained calcium influx via site-specific tyrosine phosphorylation of the phospholipase Cgamma Src homology 2-Src homology 3 linker. J. Biol. Chem..

[84] Shillitoe B., Gennery A. (2017). X-Linked Agammaglobulinaemia: Outcomes in the modern era. Clin Immunol..

[85] Pal Singh S., Dammeijer F., Hendriks R.W. (2018). Role of Bruton’s tyrosine kinase in B cells and malignancies. Mol. Cancer.

[86] Martin E., Aigrot M.S., Grenningloh R., Stankoff B., Lubetzki C., Boschert U., Zalc B. (2020). Bruton’s Tyrosine Kinase Inhibition Promotes Myelin Repair. Brain Plasticity.

[87] Keaney J., Gasser J., Gillet G., Scholz D., Kadiu I. (2019). Inhibition of Bruton’s Tyrosine Kinase Modulates Microglial Phagocytosis: Therapeutic Implications for Alzheimer’s Disease. J. Neuroimmune Pharmacol..

[88] Glendenning L., Gruber R., Dufault M., Chretien N., Proto J., Zhang M., Lamorte M., Havari E., Turner T., Chomyk A. (2020). Decoding Bruton’s tyrosine kinase signalling in neuroinflammation. MSVirtual.

[89] Nam H.Y., Nam J.H., Yoon G., Lee J.Y., Nam Y., Kang H.J., Cho H.J., Kim J., Hoe H.S. (2018). Ibrutinib suppresses LPS-induced neuroinflammatory responses in BV2 microglial cells and wild-type mice. J. Neuroinflamm..

[90] Menzfeld C., John M., van Rossum D., Regen T., Scheffel J., Janova H., Gotz A., Ribes S., Nau R., Borisch A. (2015). Tyrphostin AG126 exerts neuroprotection in CNS inflammation by a dual mechanism. Glia.

[91] Shideman C.R., Hu S., Peterson P.K., Thayer S.A. (2006). CCL5 evokes calcium signals in microglia through a kinase-, phosphoinositide-, and nucleotide-dependent mechanism. J. Neurosci. Res..

[92] Lin H., Lee E., Hestir K., Leo C., Huang M., Bosch E., Halenbeck R., Wu G., Zhou A., Behrens D. (2008). Discovery of a cytokine and its receptor by functional screening of the extracellular proteome. Science.

[93] Stanley E.R., Heard P.M. (1977). Factors regulating macrophage production and growth. Purification and some properties of the colony stimulating factor from medium conditioned by mouse L cells. J. Biol. Chem..

[94] Stanley E.R., Chitu V. (2014). CSF-1 receptor signaling in myeloid cells. Cold Spring Harb. Perspect. Biol..

[95] Elmore M.R., Najafi A.R., Koike M.A., Dagher N.N., Spangenberg E.E., Rice R.A., Kitazawa M., Matusow B., Nguyen H., West B.L. (2014). Colony-stimulating factor 1 receptor signaling is necessary for microglia viability, unmasking a microglia progenitor cell in the adult brain. Neuron.

[96] Hawley C.A., Rojo R., Raper A., Sauter K.A., Lisowski Z.M., Grabert K., Bain C.C., Davis G.M., Louwe P.A., Ostrowski M.C. (2018). Csf1r-mApple Transgene Expression and Ligand Binding In Vivo Reveal Dynamics of CSF1R Expression within the Mononuclear Phagocyte System. J. Immunol..

[97] Chitu V., Gokhan S., Nandi S., Mehler M.F., Stanley E.R. (2016). Emerging Roles for CSF-1 Receptor and its Ligands in the Nervous System. Trends Neurosci..

[98] Konno T., Yoshida K., Mizuno T., Kawarai T., Tada M., Nozaki H., Ikeda S.I., Nishizawa M., Onodera O., Wszolek Z.K. (2017). Clinical and genetic characterization of adult-onset leukoencephalopathy with axonal spheroids and pigmented glia associated with CSF1R mutation. Eur. J. Neurol..

[99] Erblich B., Zhu L., Etgen A.M., Dobrenis K., Pollard J.W. (2011). Absence of colony stimulation factor-1 receptor results in loss of microglia, disrupted brain development and olfactory deficits. PLoS ONE.

[100] Hagan N., Kane J.L., Grover D., Woodworth L., Madore C., Saleh J., Sancho J., Liu J., Li Y., Proto J. (2020). CSF1R signaling is a regulator of pathogenesis in progressive MS. Cell Death Dis..

[101] Nissen J.C., Thompson K.K., West B.L., Tsirka S.E. (2018). Csf1R inhibition attenuates experimental autoimmune encephalomyelitis and promotes recovery. Exp. Neurol..

[102] Lei F., Cui N., Zhou C., Chodosh J., Vavvas D.G., Paschalis E.I. (2020). CSF1R inhibition by a small-molecule inhibitor is not microglia specific; affecting hematopoiesis and the function of macrophages. Proc. Natl. Acad. Sci. USA.

[103] Tahmasebi F., Pasbakhsh P., Mortezaee K., Madadi S., Barati S., Kashani I.R. (2019). Effect of the CSF1R inhibitor PLX3397 on remyelination of corpus callosum in a cuprizone-induced demyelination mouse model. J. Cell Biochem..

[104] Beckmann N., Giorgetti E., Neuhaus A., Zurbruegg S., Accart N., Smith P., Perdoux J., Perrot L., Nash M., Desrayaud S. (2018). Brain region-specific enhancement of remyelination and prevention of demyelination by the CSF1R kinase inhibitor BLZ945. Acta Neuropathol. Commun..

[105] Luo J., Elwood F., Britschgi M., Villeda S., Zhang H., Ding Z., Zhu L., Alabsi H., Getachew R., Narasimhan R. (2013). Colony-stimulating factor 1 receptor (CSF1R) signaling in injured neurons facilitates protection and survival. J. Exp. Med..

[106] Ulland T.K., Colonna M. (2018). TREM2—A key player in microglial biology and Alzheimer disease. Nat. Rev. Neurol..

[107] Paloneva J., Manninen T., Christman G., Hovanes K., Mandelin J., Adolfsson R., Bianchin M., Bird T., Miranda R., Salmaggi A. (2002). Mutations in two genes encoding different subunits of a receptor signaling complex result in an identical disease phenotype. Am. J. Hum. Genet..

[108] Jonsson T., Stefansson H., Steinberg S., Jonsdottir I., Jonsson P.V., Snaedal J., Bjornsson S., Huttenlocher J., Levey A.I., Lah J.J. (2013). Variant of TREM2 associated with the risk of Alzheimer’s disease. N. Engl. J. Med..

[109] Cuyvers E., Bettens K., Philtjens S., Van Langenhove T., Gijselinck I., van der Zee J., Engelborghs S., Vandenbulcke M., Van Dongen J., Geerts N. (2014). Investigating the role of rare heterozygous TREM2 variants in Alzheimer’s disease and frontotemporal dementia. Neurobiol. Aging.

[110] Mittelbronn M., Dietz K., Schluesener H.J., Meyermann R. (2001). Local distribution of microglia in the normal adult human central nervous system differs by up to one order of magnitude. Acta Neuropathol..

[111] Forabosco P., Ramasamy A., Trabzuni D., Walker R., Smith C., Bras J., Levine A.P., Hardy J., Pocock J.M., Guerreiro R. (2013). Insights into TREM2 biology by network analysis of human brain gene expression data. Neurobiol. Aging.

[112] Wunderlich P., Glebov K., Kemmerling N., Tien N.T., Neumann H., Walter J. (2013). Sequential proteolytic processing of the triggering receptor expressed on myeloid cells-2 (TREM2) protein by ectodomain shedding and gamma-secretase-dependent intramembranous cleavage. J. Biol. Chem..

[113] Takahashi K., Rochford C.D., Neumann H. (2005). Clearance of apoptotic neurons without inflammation by microglial triggering receptor expressed on myeloid cells-2. J. Exp. Med..

[114] Takahashi K., Prinz M., Stagi M., Chechneva O., Neumann H. (2007). TREM2-transduced myeloid precursors mediate nervous tissue debris clearance and facilitate recovery in an animal model of multiple sclerosis. PLoS Med..

[115] Cignarella F., Filipello F., Bollman B., Cantoni C., Locca A., Mikesell R., Manis M., Ibrahim A., Deng L., Benitez B.A. (2020). TREM2 activation on microglia promotes myelin debris clearance and remyelination in a model of multiple sclerosis. Acta Neuropathol..

[116] Bouchon A., Hernandez-Munain C., Cella M., Colonna M. (2001). A DAP12-mediated pathway regulates expression of CC chemokine receptor 7 and maturation of human dendritic cells. J. Exp. Med..

[117] Kobayashi M., Konishi H., Sayo A., Takai T., Kiyama H. (2016). TREM2/DAP12 Signal Elicits Proinflammatory Response in Microglia and Exacerbates Neuropathic Pain. J. Neurosci..

[118] Harrison J.K., Jiang Y., Chen S., Xia Y., Maciejewski D., McNamara R.K., Streit W.J., Salafranca M.N., Adhikari S., Thompson D.A. (1998). Role for neuronally derived fractalkine in mediating interactions between neurons and CX3CR1-expressing microglia. Proc. Natl. Acad. Sci. USA.

[119] Lucas A.D., Chadwick N., Warren B.F., Jewell D.P., Gordon S., Powrie F., Greaves D.R. (2001). The transmembrane form of the CX3CL1 chemokine fractalkine is expressed predominantly by epithelial cells in vivo. Am. J. Pathol.

[120] Limatola C., Ransohoff R.M. (2014). Modulating neurotoxicity through CX3CL1/CX3CR1 signaling. Front. Cell Neurosci..

[121] Zujovic V., Benavides J., Vige X., Carter C., Taupin V. (2000). Fractalkine modulates TNF-alpha secretion and neurotoxicity induced by microglial activation. Glia.

[122] Mizuno T., Kawanokuchi J., Numata K., Suzumura A. (2003). Production and neuroprotective functions of fractalkine in the central nervous system. Brain Res..

[123] Murai N., Mitalipova M., Jaenisch R. (2020). Functional analysis of CX3CR1 in human induced pluripotent stem (iPS) cell-derived microglia-like cells. Eur. J. Neurosci..

[124] Lee S., Varvel N.H., Konerth M.E., Xu G., Cardona A.E., Ransohoff R.M., Lamb B.T. (2010). CX3CR1 deficiency alters microglial activation and reduces beta-amyloid deposition in two Alzheimer’s disease mouse models. Am. J. Pathol..

[125] Liu Z., Condello C., Schain A., Harb R., Grutzendler J. (2010). CX3CR1 in microglia regulates brain amyloid deposition through selective protofibrillar amyloid-beta phagocytosis. J. Neurosci..

[126] Sunnemark D., Eltayeb S., Nilsson M., Wallstrom E., Lassmann H., Olsson T., Berg A.L., Ericsson-Dahlstrand A. (2005). CX3CL1 (fractalkine) and CX3CR1 expression in myelin oligodendrocyte glycoprotein-induced experimental autoimmune encephalomyelitis: Kinetics and cellular origin. J. Neuroinflamm..

[127] Lauro C., Cipriani R., Catalano M., Trettel F., Chece G., Brusadin V., Antonilli L., van Rooijen N., Eusebi F., Fredholm B.B. (2010). Adenosine A1 receptors and microglial cells mediate CX3CL1-induced protection of hippocampal neurons against Glu-induced death. Neuropsychopharmacology.

[128] Cipriani R., Villa P., Chece G., Lauro C., Paladini A., Micotti E., Perego C., De Simoni M.G., Fredholm B.B., Eusebi F. (2011). CX3CL1 is neuroprotective in permanent focal cerebral ischemia in rodents. J. Neurosci..

[129] Catalano M., Lauro C., Cipriani R., Chece G., Ponzetta A., Di Angelantonio S., Ragozzino D., Limatola C. (2013). CX3CL1 protects neurons against excitotoxicity enhancing GLT-1 activity on astrocytes. J. Neuroimmunol..

[130] Garcia J.A., Pino P.A., Mizutani M., Cardona S.M., Charo I.F., Ransohoff R.M., Forsthuber T.G., Cardona A.E. (2013). Regulation of adaptive immunity by the fractalkine receptor during autoimmune inflammation. J. Immunol..

[131] Mecca C., Giambanco I., Donato R., Arcuri C. (2018). Microglia and Aging: The Role of the TREM2-DAP12 and CX3CL1-CX3CR1 Axes. Int. J. Mol. Sci..

[132] Imai T., Hieshima K., Haskell C., Baba M., Nagira M., Nishimura M., Kakizaki M., Takagi S., Nomiyama H., Schall T.J. (1997). Identification and molecular characterization of fractalkine receptor CX3CR1, which mediates both leukocyte migration and adhesion. Cell.

[133] Infante-Duarte C., Weber A., Kratzschmar J., Prozorovski T., Pikol S., Hamann I., Bellmann-Strobl J., Aktas O., Dorr J., Wuerfel J. (2005). Frequency of blood CX3CR1-positive natural killer cells correlates with disease activity in multiple sclerosis patients. FASEB J..

[134] Ridderstad Wollberg A., Ericsson-Dahlstrand A., Jureus A., Ekerot P., Simon S., Nilsson M., Wiklund S.J., Berg A.L., Ferm M., Sunnemark D. (2014). Pharmacological inhibition of the chemokine receptor CX3CR1 attenuates disease in a chronic-relapsing rat model for multiple sclerosis. Proc. Natl. Acad. Sci. USA.

[135] Tsuda M., Shigemoto-Mogami Y., Koizumi S., Mizokoshi A., Kohsaka S., Salter M.W., Inoue K. (2003). P2X4 receptors induced in spinal microglia gate tactile allodynia after nerve injury. Nature.

[136] Agresti C., Meomartini M.E., Amadio S., Ambrosini E., Serafini B., Franchini L., Volonte C., Aloisi F., Visentin S. (2005). Metabotropic P2 receptor activation regulates oligodendrocyte progenitor migration and development. Glia.

[137] Khoja S., Huynh N., Asatryan L., Jakowec M.W., Davies D.L. (2018). Reduced expression of purinergic P2X4 receptors increases voluntary ethanol intake in C57BL/6J mice. Alcohol.

[138] Wyatt L.R., Godar S.C., Khoja S., Jakowec M.W., Alkana R.L., Bortolato M., Davies D.L. (2013). Sociocommunicative and sensorimotor impairments in male P2X4-deficient mice. Neuropsychopharmacology.

[139] Vazquez-Villoldo N., Domercq M., Martin A., Llop J., Gomez-Vallejo V., Matute C. (2014). P2X4 receptors control the fate and survival of activated microglia. Glia.

[140] Masuda T., Iwamoto S., Yoshinaga R., Tozaki-Saitoh H., Nishiyama A., Mak T.W., Tamura T., Tsuda M., Inoue K. (2014). Transcription factor IRF5 drives P2X4R+-reactive microglia gating neuropathic pain. Nat. Commun..

[141] Krausgruber T., Blazek K., Smallie T., Alzabin S., Lockstone H., Sahgal N., Hussell T., Feldmann M., Udalova I.A. (2011). IRF5 promotes inflammatory macrophage polarization and TH1-TH17 responses. Nat. Immunol..

[142] Tolwani R.J., Cosgaya J.M., Varma S., Jacob R., Kuo L.E., Shooter E.M. (2004). BDNF overexpression produces a long-term increase in myelin formation in the peripheral nervous system. J. Neurosci. Res..

[143] Su W.F., Wu F., Jin Z.H., Gu Y., Chen Y.T., Fei Y., Chen H., Wang Y.X., Xing L.Y., Zhao Y.Y. (2019). Overexpression of P2X4 receptor in Schwann cells promotes motor and sensory functional recovery and remyelination via BDNF secretion after nerve injury. Glia.

[144] Zabala A., Vazquez-Villoldo N., Rissiek B., Gejo J., Martin A., Palomino A., Perez-Samartin A., Pulagam K.R., Lukowiak M., Capetillo-Zarate E. (2018). P2X4 receptor controls microglia activation and favors remyelination in autoimmune encephalitis. EMBO Mol. Med..

[145] Jurga A.M., Piotrowska A., Makuch W., Przewlocka B., Mika J. (2017). Blockade of P2X4 Receptors Inhibits Neuropathic Pain-Related Behavior by Preventing MMP-9 Activation and, Consequently, Pronociceptive Interleukin Release in a Rat Model. Front. Pharmacol..

[146] Domercq M., Matute C. (2019). Targeting P2X4 and P2X7 receptors in multiple sclerosis. Curr. Opin. Pharmacol..

[147] Ledderose C., Liu K., Kondo Y., Slubowski C.J., Dertnig T., Denicolo S., Arbab M., Hubner J., Konrad K., Fakhari M. (2018). Purinergic P2X4 receptors and mitochondrial ATP production regulate T cell migration. J. Clin. Investig..

[148] Daneman R., Prat A. (2015). The blood-brain barrier. Cold Spring Harb. Perspect. Biol..

[149] Hersh D.S., Wadajkar A.S., Roberts N., Perez J.G., Connolly N.P., Frenkel V., Winkles J.A., Woodworth G.F., Kim A.J. (2016). Evolving Drug Delivery Strategies to Overcome the Blood Brain Barrier. Curr. Pharm. Des..

[150] Zhao N., Francis N.L., Calvelli H.R., Moghe P.V. (2020). Microglia-targeting nanotherapeutics for neurodegenerative diseases. APL Bioeng..

